# LEADER 5: prevalence and cardiometabolic impact of obesity in cardiovascular high-risk patients with type 2 diabetes mellitus: baseline global data from the LEADER trial

**DOI:** 10.1186/s12933-016-0341-5

**Published:** 2016-02-10

**Authors:** L. Masmiquel, L. A. Leiter, J. Vidal, S. Bain, J. Petrie, E. Franek, I. Raz, A. Comlekci, S. Jacob, L. van Gaal, F. M. M. Baeres, S. P. Marso, M. Eriksson, on behalf of the LEADER investigators

**Affiliations:** Endocrinology and Nutrition Department, Hospital Son Llàtzer, University Institute of Health Science Research (IUNICS-IdISPa), Universitat de les Illes Balears, Palma, Majorca, Spain; Divisions of Endocrinology and Metabolism, Li Ka Shing Knowledge Institute and Keenan Research Centre for Biomedical Science, St. Michael’s Hospital, University of Toronto, Toronto, ON Canada; Endocrinology and Nutrition Department, Hospital Clínic, Institut d’Investigacions Biomèdiques August Pi i Sunyer (IDIBAPS), University of Barcelona, Barcelona, Spain; Institute of Life Science, Swansea University, Swansea, UK; Institute of Cardiovascular and Medical Science, BHF Glasgow Cardiovascular Research Centre, University of Glasgow, Glasgow, UK; Mossakowski Medical Research Centre, Polish Academy of Sciences and Central Clinical Hospital MSW, Warsaw, Poland; Diabetes Unit, Internal Medicine Division, Hadassah Hebrew University Hospital, Jerusalem, Israel; Department of Internal Medicine, Division of Endocrinology, Inciralti, Izmir, Turkey; Praxis für Prävention und Therapie, Kardio Metabolischen Instituts, Villingen-Schwenningen, Germany; Department of Endocrinology, Diabetology, and Metabolism, Faculty of Medicine, Antwerp University Hospital, Edegem, Antwerp, Belgium; Novo Nordisk, Copenhagen, Denmark; Division of Cardiology, University of Texas Southwestern Medical Center, Dallas, TX USA; Department of Endocrinology, Metabolism and Diabetes, Karolinska University Hospital, Stockholm, Sweden

**Keywords:** Liraglutide, Cardiovascular, Type 2 diabetes, LEADER, Cardiometabolic, Obesity, Overweight

## Abstract

**Background:**

Epidemiological data on obesity are needed, particularly in patients with type 2 diabetes mellitus (T2DM) and high cardiovascular (CV) risk. We used the baseline data of liraglutide effect and action in diabetes: evaluation of CV outcome results—A long term Evaluation (LEADER) (a clinical trial to assess the CV safety of liraglutide) to investigate: (i) prevalence of overweight and obesity; (ii) relationship of the major cardiometabolic risk factors with anthropometric measures of adiposity [body mass index (BMI) and waist circumference (WC)]; and (iii) cardiometabolic treatment intensity in relation to BMI and WC.

**Methods:**

LEADER enrolled two distinct populations of high-risk patients with T2DM in 32 countries: (1) aged ≥50 years with prior CV disease; (2) aged ≥60 years with one or more CV risk factors. Associations of metabolic variables, demographic variables and treatment intensity with anthropometric measurements (BMI and WC) were explored using regression models (ClinicalTrials.gov identifier: NCT01179048).

**Results:**

Mean BMI was 32.5 ± 6.3 kg/m^2^ and only 9.1 % had BMI <25 kg/m^2^. The prevalence of healthy WC was also extremely low (6.4 % according to International Joint Interim Statement for the Harmonization of the Metabolic Syndrome criteria). Obesity was associated with being younger, female, previous smoker, Caucasian, American, with shorter diabetes duration, uncontrolled blood pressure (BP), antihypertensive agents, insulin plus oral antihyperglycaemic treatment, higher levels of triglycerides and lower levels of high-density lipoprotein cholesterol.

**Conclusions:**

Overweight and obesity are prevalent in high CV risk patients with T2DM. BMI and WC are related to the major cardiometabolic risk factors. Furthermore, treatment intensity, such as insulin, statins or oral antihypertensive drugs, is higher in those who are overweight or obese; while BP and lipid control in these patients are remarkably suboptimal. LEADER confers a unique opportunity to explore the longitudinal effect of weight on CV risk factors and hard endpoints.

## Background

The association between different anthropometric measures of adiposity and cardiovascular (CV) risk in patients with established diabetes and high CV risk has not been well studied [1, 2]. Several longitudinal cohort studies have shown that patients with coronary artery disease and type 2 diabetes mellitus (T2DM) have lower mortality with a higher body mass index (BMI), suggesting that an “obesity paradox” exists [3]. Although lower muscle mass in patients with T2DM could be a plausible biological explanation for this [4], several confounding factors are likely to play a role in this apparent paradox. For example, BMI alone is recognised to be an imperfect measurement of the type of adiposity. Furthermore, some imbalance could be evident in other key parameters, including age, lipid levels, degree of metabolic control, CV risk factors and treatment intensity [5, 6]. Therefore, additional data on the associations of these parameters are needed, especially in high CV risk patients with T2DM.

The LEADER (liraglutide effect and action in diabetes: evaluation of CV outcome results) trial is designed to assess formally the CV safety of liraglutide in patients with T2DM aged ≥50 years with history of, or at high risk for CV events [7]. We used the cross-sectional baseline data from LEADER to investigate (i) the prevalence of overweight and obesity; (ii) the interplay among the major cardiometabolic risk factors and two anthropometric measures of adiposity [BMI and waist circumference (WC)]; and (iii) the treatment of cardiometabolic risk factors in relation to BMI and WC categories. Furthermore, because race and ethnicity significantly affect the associations between anthropometric indices and CV risk factors [8–10], we also investigated the influence of these characteristics.

## Methods

LEADER is a phase 3B, multicentre, international, randomised, double-blind, placebo-controlled clinical trial with long-term follow-up designed to assess the CV safety of liraglutide up to 1.8 mg daily, a glucagon-like peptide-1 receptor agonist (GLP-1RA) approved for treatment of patients with T2DM. A detailed description of the study design and baseline characteristics of the study population has previously been published [7]. Briefly, the trial enrolled a CV high-risk population of patients with T2DM at 410 sites in 32 countries who fit into one of the following categories: i) patients >50 years of age and concomitant CV disease (CVD), cerebrovascular disease, peripheral vascular disease, chronic renal failure, or chronic heart failure; ii) patients >60 years of age and one or more of the following CV risk factors: microalbuminuria or proteinuria, hypertension and left ventricular hypertrophy by electrocardiogram or imaging, left ventricular dysfunction by imaging, or ankle–brachial index of <0.9.

Physical exploration and anthropometric measurements were carried out according to the protocol by the investigators with the subject barefoot and wearing light clothes. BMI was the ratio of weight (kg) divided by squared height (m^2^). WC was measured from the front at the narrowest point between the rib cage and the iliac crest when the subject was breathing out gently.

Smoking habits, comorbidities and race/ethnicity were documented at study entry. Ethnicity was obtained separately from race. The ethnicity was classified as hispanic/latino or not hispanic/latino; the race was classified as Caucasian, Black, Asian, or Other.

All subjects gave written informed consent and the trial was approved by the relevant local ethical committees and was conducted in conformity with the declaration of Helsinki.

### Statistical analysis

Data are presented as mean (standard deviation), median (interquartile range) or number (%). To examine the categorical relationship between BMI and the clinical, epidemiological and biochemical variables, patients were divided into the following categories based on their BMI (kg/m^2^): <25.0 (healthy weight), 25.0 to <30 (overweight), 30.0 to <35.0 (obesity grade I), 35.0 to <40 (obesity grade II), ≥40.0 (obesity grade III) [11]. For the same purpose, WC was dichotomised as healthy/unhealthy for both the National Cholesterol Education Program [adult treatment panel III criteria (ATPIII)] [12] (≥88 cm for females and ≥102 cm for males) and the International Joint Interim Statement for the Harmonization of the Metabolic Syndrome criteria (IISHMS) [13] (Caucasians, Blacks and Others: ≥94 cm males and ≥80 cm females; Hispanics and Asians: ≥90 cm males and ≥80 cm females) [14].

For statistical purposes, the percentage of patients with glycated haemoglobin (HbA1c) ≤8 % (<63.9 mmol/mol) was calculated as it is considered to be an appropriate target in populations with advanced micro- or macrovascular complications, and extensive comorbid conditions, and in those with long-standing diabetes in whom the general target of an HbA1c <7.0 % (<53.0 mmol/mol) is difficult to attain [15, 16]. Blood pressure (BP) measurements of <140/80 mmHg, low-density lipoprotein cholesterol (LDL-C) <2.6 mmol/l (100 mg/dl) (<1.8 mmol/l [70 mg/dl] in patients with previous CV events), high-density lipoprotein cholesterol (HDL-C) >1.3 mmol/l (50 mg/dl) in women and >1.0 mmol/l (40 mg/dl) in men and triglycerides <1.7 mmol/l (150 mg/dl) were considered to be at target, according to the American Diabetes Association 2014 guidelines (the guidelines available when this analysis was conducted) [15].

Associations between BMI or WC with covariates and factors were screened initially using Spearman correlation and Chi square or Fisher’s exact test, respectively. Linear regression models were used to describe the associations of treatment intensity/metabolic variables with anthropometric measurements. Likewise, logistic regression models were developed to assess the association between dichotomised “off-target” anthropometric variables and explanatory factors. Variable selection was decided before model fitting; in particular, BMI was excluded from the analysis of WC and vice versa (an overview of variables used in the logistic regression model is provided in the data table). Covariates were selected by consideration of potential impact on anthropometric measurements, based on review of the literature and availability in the LEADER database. Main effects of all covariates/factors were retained in the model, as the sample size of the LEADER study implied no need for further variable selection.

This study is based on the data from all 9340 participants in the LEADER trial, but numbers may vary due to missing data for a small number of participants. Complete data are available for approximately 99 % of participants. Individuals with missing data were not included in the statistical analyses.

A two-sided p value of <0.01 was considered statistically significant. Statistical analyses were performed using SAS 9.3 software (SAS Institute, Cary, NC, USA). Analyses were conducted on the data available at baseline.

## Results

### Demographic and clinical characteristics

Baseline characteristics of all randomised patients stratified by prior CVD, BMI and WC are detailed in Tables 1–3.

**Table 1:** Demographic and clinical characteristics stratified by CVD

	All	No prior CVD group	Prior CVD group
	(n = 9340)	(n = 1748)	(n = 7592)
Age (years)	64.3 ± 7.2	65.8 ± 5.2	63.9 ± 7.6
Gender			
Female	3337 (35.7 %)	793 (45.4 %)	2544 (33.5 %)
Male	6003 (64.3 %)	955 (54.6 %)	5048 (66.5 %)
Age group			
50–59 years	2321 (24.9 %)	26 (1.5 %)	2295 (30.2 %)
60–69 years	4839 (51.8 %)	1337 (76.5 %)	3502 (46.1 %)
70–79 years	1977 (21.2 %)	359 (20.5 %)	1618 (21.3 %)
80–89 years	199 (2.1 %)	26 (1.5 %)	173 (2.3 %)
90–99 years	4 (0.0 %)	0 (0.0 %)	4 (0.1 %)
BMI (kg/m^2^)			
<25.0	865 (9.3 %)	172 (9.8 %)	693 (9.1 %)
25 to <30	2671 (28.6 %)	520 (29.7 %)	2151 (28.3 %)
30 to <35	2987 (32.0 %)	535 (30.6 %)	2452 (32.3 %)
35 to <40	1715 (18.4 %)	310 (17.7 %)	1405 (18.5 %)
≥40.0	1092 (11.7 %)	210 (12.0 %)	882 (11.6 %)
WC ATIII target			
Yes	1933 (20.7 %)	338 (19.3 %)	1595 (21.0 %)
No	7330 (78.5 %)	1398 (80.0 %)	5932 (78.1 %)
Missing values	77 (0.8 %)	12 (0.7 %)	65 (0.9 %)
WC-IISHMS target			
Yes	585 (6.3 %)	100 (5.7 %)	485 (6.4 %)
No	8678 (92.9 %)	1636 (93.6 %)	7042 (92.8 %)
Missing values	77 (0.8)	12 (0.7)	65 (0.9 %)
HbA1c (%)	8.7 (1.6)	8.8 (1.5)	8.7 (1.6)
HbA1c (mmol/mol)	71.6 (17.5)	72.7 (16.4)	71.6 (17.5)
Blood lipids			
LDL-C (mmol/L)	2.3 (0.9)	2.5 (0.9)	2.3 (0.9)
HDL-C (mmol/L)	1.2 (0.3)	1.2 (0.3)	1.2 (0.3)
Triglycerides (mmol/L)	2.1 (1.6)	2.0 (1.5)	2.1 (1.6)
No OAD use at baseline	4409 (47.2 %)	744 (42.6 %)	3665 (48.3 %)
Pre-treatment			
None/diet	504 (5.4)	99 (5.7)	405 (5.3)
Insulines only	665 (7.1)	69 (3.9)	596 (7.9)
OADs only	4931 (52.8)	1004 (57.4)	3927 (51.7)
Ins + OADs	3240 (34.7)	576 (33.0)	2644 (35.1)
Use of antihypertensive medication			
Yes	8550 (91.5 %)	1473 (84.3 %)	7077 (93.2 %)
No	790 (8.5 %)	275 (15.7 %)	515 (6.8 %)

Values are expressed as mean ± SD or frequency (percent of row). *ATPIII* Adult treatment panel III criteria; *CVD* cardiovascular disease; *eGFR* estimated glomerular filtration rate; *HbA1c* glycated haemoglobin; *HDL-C* high-density lipoprotein cholesterol; *IISHMS* International Joint Interim Statement for the Harmonization of the Metabolic Syndrome criteria; *LDL-C* low-density lipoprotein cholesterol; *SD* standard deviation; *WC* waist circumference

### Stratified by CVD

Mean BMI was comparable between patients with prior CVD and those without (32.5 vs. 32.4 kg/m^2^, respectively). Comparing patients with prior CVD with those without, the prevalence of overweight (28.3 vs. 29.7 %, respectively), obesity grade I (32.3 vs. 30.6 %, respectively), obesity grade II (18.5 vs. 17.7 %, respectively), and obesity grade III (11.6 vs. 12.0 %, respectively) was similar (Table 1). Likewise, comparing patients with prior CVD with those without mean WC (110.1 and 109.4 cm, respectively) and the overall prevalence of abdominal obesity according to ATPIII (78.1 and 80.0 %, respectively) and IISHMS (92.8 and 93.6 %, respectively) was also similar.

Female gender was less prevalent in the prior CVD cohort than those without prior CVD (33.5 vs. 45.4 %, respectively). There was also a lower prevalence of patients aged 60–69 years with prior CVD than patients without (46.1 vs. 76.5 %, respectively), and a higher proportion of patients aged 50–59 years with prior CVD than patients without (30.2 vs. 1.5 %, respectively). Furthermore, there was also a lower prevalence of patients at target LDL-C with prior CVD than those without (33.2 vs. 56.6 %, respectively). Comparing patients with prior CVD with those without, there was a greater prevalence of patients not using oral antihyperglycaemic drugs (OADs) (48.3 vs. 42.6 %, respectively). Additionally, comparing patients with prior CVD with those without, there was a greater prevalence of patients using antihypertensive medication (93.2 vs. 84.3 %, respectively).

### Stratified by BMI and WC

Mean BMI was 32.5 ± 6.3 kg/m^2^ and only 9.1 % of patients had a BMI <25 kg/m^2^. The prevalence of overweight, obesity grade I, obesity grade II, and obesity grade III was 28.6, 32.0, 18.4 and 11.7 %, respectively (Table 2). The mean WC was 109.9 ± 16.2 cm and the overall prevalence of abdominal obesity according to ATPIII and IISHMS was 79.1 and 93.6 %, respectively (Table 3).

**Table 2:** Demographic and clinical characteristics stratified by BMI

	All	<25 kg/m^2^	25 to <30 kg/m^2^	30 to <35 kg/m^2^	35 to <40 kg/m^2^	≥40.0 kg/m^2^
	(n = 9330)	(n = 865)	(n = 2671)	(n = 2987)	(n = 1715)	(n = 1092)
Age (years)	64.3 ± 7.2	64.7 ± 7.7	65.4 ± 7.4	64.4 ± 7.2	63.4 ± 6.8	62.5 ± 6.6
Gender						
Female	3331	262 (7.9 %)	817 (24.5 %)	995 (29.9 %)	706 (21.2 %)	551 (16.5 %)
Male	5999	603 (10.1 %)	1854 (30.9 %)	1992 (33.2 %)	1009 (16.8 %)	541 (9.0 %)
Age group						
50–59 years	2317	201 (8.7 %)	566 (24.4 %)	729 (31.5 %)	476 (20.5 %)	345 (14.9 %)
60–69 years	4834	435 (9.0 %)	1327 (27.5 %)	1548 (32.0 %)	922 (19.1 %)	602 (12.5 %)
70–79 years	1976	201 (10.2 %)	697 (35.3 %)	646 (32.7 %)	298 (15.1 %)	135 (6.8 %)
80–89 years	199	26 (13.1 %)	80 (40.2 %)	64 (32.2 %)	19 (9.5 %)	10 (5.0 %)
90–99 years	4	3 (75.0 %)	1 (25.0 %)	0 (0.0 %)	0 (0.0 %)	0 (0.0 %)
Region						
Europe	3517	206 (5.9 %)	1019 (29.0 %)	1240 (35.3 %)	667 (19.0 %)	385 (10.9 %)
Other areas	2616	276 (10.6 %)	807 (30.8 %)	830 (31.7 %)	457 (17.5 %)	246 (9.4 %)
United States	2487	120 (4.8 %)	529 (21.3 %)	811 (32.6 %)	569 (22.9 %)	458 (18.4 %)
Asia	710	263 (37.0 %)	316 (44.5 %)	106 (14.9 %)	22 (3.1 %)	3 (0.4 %)
Race						
Asian	920	326 (35.4 %)	395 (42.9 %)	159 (17.3 %)	33 (3.6 %)	7 (0.8 %)
Black	773	53 (6.9 %)	225 (29.1 %)	227 (29.4 %)	155 (20.1 %)	113 (14.6 %)
Other	406	57 (14.0 %)	150 (36.9 %)	118 (29.1 %)	50 (12.3 %)	31 (7.6 %)
Caucasian	7231	429 (5.9 %)	1901 (26.3 %)	2483 (34.3 %)	1477 (20.4 %)	941 (13.0 %)
Ethnicity						
Hispanic or latino	1135	131 (11.5 %)	393 (34.6 %)	357 (31.5 %)	166 (14.6 %)	88 (7.8 %)
Not hispanic or latino	8195	734 (9.0 %)	2278 (27.8 %)	2630 (32.1 %)	1549 (18.9 %)	1004 (12.3 %)
eGFR category (mL/min/1.73 m^2^)						
Normal (≥90)	3445	367 (10.7 %)	983 (28.5 %)	1050 (30.5 %)	670 (19.4 %)	375 (10.9 %)
Mild (60–90)	3854	304 (7.9 %)	1119 (29.0 %)	1299 (33.7 %)	694 (18.0 %)	438 (11.4 %)
Moderate (30–60)	1852	171 (9.2 %)	510 (27.5 %)	600 (32.4 %)	322 (17.4 %)	249 (13.4 %)
Severe (<30)	177	23 (13.0 %)	58 (32.8 %)	37 (20.9 %)	29 (16.4 %)	30 (16.9 %)
Smoker						
Current smoker	1128	122 (10.8 %)	351 (31.1 %)	359 (31.8 %)	174 (15.4 %)	122 (10.8 %)
Never smoked	3867	422 (10.9 %)	1108 (28.7 %)	1172 (30.3 %)	718 (18.6 %)	447 (11.6 %)
Previous smoker	4335	321 (7.4 %)	1212 (28.0 %)	1456 (33.6 %)	826 (19.1 %)	523 (12.1 %)
CVD stratum						
No Prior CVD group	1747	172 (9.8 %)	520 (29.8 %)	535 (30.6 %)	310 (17.7 %)	210 (12.0 %)
Prior CVD group	7583	693 (9.1 %)	2151 (28.4 %)	2452 (32.3 %)	1405 (18.5 %)	882 (11.6 %)
Diabetes duration (years)	12.7 ± 9.5	14.1 ± 8.8	13.5 ± 8.2	12.4 ± 7.8	12.0 ± 7.7	12.1 ± 7.7
HbA1c (%)	8.7 ± 1.5	8.9 ± 1.8	8.6 ± 1.5	8.6 ± 1.5	8.7 ± 1.5	8.7 ± 1.5
HbA1c (mmol/mol)	(71.6 ± 16.4)	(73.8 ± 19.7)	(70.5 ± 16.4)	(70.5 ± 16.4)	(71.6 ± 16.4)	(71.6 ± 16.4)
Blood lipids						
LDL-C (mmol/L)	2.3 ± 0.9	2.3 ± 0.5	2.3 ± 0.9	2.3 ± 0.9	2.3 ± 0.9	2.3 ± 0.9
HDL-C (mmol/L)	1.2 ± 0.3	1.2 ± 0.4	1.2 ± 0.3	1.1 ± 0.3	1.2 ± 0.3	1.2 ± 0.3
Triglycerides (mmol/L)	2.1 ± 1.6	1.6 ± 1.1	1.9 ± 1.5	2.2 ± 1.8	2.2 ± 1.4	2.2 ± 1.7
Blood pressure						
Systolic blood pressure (mmHg)	137.7 ± 18.6	134.5 ± 19.2	137.9 ± 18.5	138.3 ± 18.5	138.1 ± 18.2	137.5 ± 19.3
Diastolic blood pressure (mmHg)	77.9 ± 10.5	76.4 ± 9.9	77.1 ± 10.2	78.3 ± 10.4	78.9 ± 10.4	78.3 ± 11.5

Values are expressed as mean ± SD or frequency (percent of row). *BMI* body mass index; *CVD* cardiovascular disease; *eGFR* estimated glomerular filtration rate; *HbA1c* glycated haemoglobin; *HDL-C* high-density lipoprotein cholesterol; *LDL-C* low-density lipoprotein cholesterol; *SD* standard deviation

**Table 3:** Demographic and clinical characteristics stratified by WC according ATPIII and IISHMS

	All	WC-ATPIII		WC-IISHMS	
		Off target	On target	Off target	On target
	(n = 9263)	(n = 7330)	(n = 1933)	(n = 8678)	(n = 585)
Age (years)	64.3 ± 7.2	64.3 ± 7.2	64.3 ± 7.4	64.3 ± 7.2	64.2 ± 7.4
Gender					
Female	3305	3050 (92.3 %)	255 (7.7 %)	3232 (97.8 %)	73 (2.2 %)
Male	5958	4280 (71.8 %)	1678 (28.2 %)	5446 (91.4 %)	512 (8.6 %)
Age group					
50–59 years	2305	1822 (79.0 %)	483 (21.0 %)	2165 (93.9 %)	140 (6.1 %)
60–69 years	4806	3835 (79.8 %)	971 (20.2 %)	4501 (93.7 %)	305 (6.3 %)
70–79 years	1954	1518 (77.7 %)	436 (22.3 %)	1829 (93.6 %)	125 (6.4 %)
80–89 years	194	153 (78.9 %)	41 (21.1 %)	180 (92.8 %)	14 (7.2 %)
90–99 years	4	2 (50.0 %)	2 (50.0 %)	3 (75.0 %)	1 (25.0 %)
Region					
Europe	3489	2873 (82.3 %)	616 (17.7 %)	3322 (95.2 %)	167 (4.8 %)
Other areas	2600	2053 (79.0 %)	547 (21.0 %)	2449 (94.2 %)	151 (5.8 %)
United States	2465	2092 (84.9 %)	373 (15.1 %)	2345 (95.1 %)	120 (4.9 %)
Asia	709	312 (44.0 %)	397 (56.0 %)	562 (79.3 %)	147 (20.7 %)
Race					
Asian	919	420 (45.7 %)	499 (54.3 %)	741 (80.6 %)	178 (19.4 %)
Black	771	632 (82.0 %)	139 (18.0 %)	725 (94.0 %)	46 (6.0 %)
Other	403	298 (73.9 %)	105 (26.1 %)	374 (92.8 %)	29 (7.2 %)
Caucasian	7170	5980 (83.4 %)	1190 (16.6 %)	6838 (95.4 %)	332 (4.6 %)
Ethnicity					
Hispanic or latino	1127	840 (74.5 %)	287 (25.5 %)	1070 (94.9 %)	57 (5.1 %)
Not hispanic or latino	8136	6490 (79.8 %)	1646 (20.2 %)	7608 (93.5 %)	528 (6.5 %)
eGFR category (mL/min/1.73 m^2^)					
Normal (≥90)	3415	2654 (77.7 %)	761 (22.3 %)	3183 (93.2 %)	232 (6.8 %)
Mild (60–90)	3831	3068 (80.1 %)	763 (19.9 %)	3590 (93.7 %)	241 (6.3 %)
Moderate (30–60)	1842	1475 (80.1 %)	367 (19.9 %)	1742 (94.6 %)	100 (5.4 %)
Severe (<30)	173	132 (76.3 %)	41 (23.7 %)	161 (93.1 %)	12 (6.9 %)
Smoker					
Current smoker	1117	833 (74.6 %)	284 (25.4 %)	1023 (91.6 %)	94 (8.4 %)
Never smoked	3852	3057 (79.4 %)	795 (20.6 %)	3611 (93.7 %)	241 (6.3 %)
Previous smoker	4294	3440 (80.1 %)	854 (19.9 %)	4044 (94.2 %)	250 (5.8 %)
CVD stratum					
No prior CVD group	1736	1398 (80.5 %)	338 (19.5 %)	1636 (94.2 %)	100 (5.8 %)
Prior CVD group	7527	5932 (78.8 %)	1595 (21.2 %)	7042 (93.6 %)	485 (6.4 %)
Diabetes duration (years)	12.7 ± 8.0	12.5 ± 8.0	13.5 ± 8.3	12.6 ± 8.0	13.9 ± 8.7
HbA1c (%)	8.7 ± 1.5	8.7 ± 1.5	8.7 ± 1.6	8.7 ± 1.5	8.7 ± 1.7
HbA1c (mmol/mol)	(71.6 ± 16.4)	(71.6 ± 16.4)	(71.6 ± 17.5)	(71.6 ± 16.4)	(71.6 ± 18.6)
Blood lipids					
LDL-C (mmol/L)	2.3 ± 0.9	2.3 ± 0.9	2.3 ± 0.9	2.3 ± 0.9	2.2 ± 0.9
HDL-C (mmol/L)	1.2 ± 0.3	1.2 ± 0.3	1.2 ± 0.3	1.2 ± 0.3	1.3 ± 0.4
Triglycerides (mmol/L)	2.1 ± 1.6	2.1 ± 1.6	1.7 ± 1.5	2.1 ± 1.6	1.6 ± 1.6
Blood pressure					
Systolic blood pressure (mmHg)	138 ± 19	138 ± 19	136 ± 19	138 ± 19	135 ± 18
Diastolic blood pressure (mmHg)	78 ± 11	78 ± 11	77 ± 10	78 ± 11	76 ± 10

Values are expressed as mean ± SD or frequency (percent of row). *ATPIII* Adult Treatment Panel III criteria; *CVD* cardiovascular disease; *eGFR* estimated glomerular filtration rate; *HbA1c* glycated haemoglobin; *HDL-C* high-density lipoprotein cholesterol; *IISHMS* International Joint Interim Statement for the Harmonization of the Metabolic Syndrome criteria; *LDL-C* low-density lipoprotein cholesterol; *SD* standard deviation; *WC* waist circumference

BMI was higher in females than in males (BMI 33.6 ± 6.8 kg/m^2^ vs. 31.9 ± 5.9 kg/m^2^, respectively; p < 0.0001). WC was lower in females than males (107.6 ± 15.6 cm vs. 111.2 ± 16.3 cm, respectively; p < 0.0001). Moreover, due to gender-specific cut-offs, the prevalence of abdominal obesity was higher in females than males when measured independently from the WC criteria (ATPIII: 92.3 vs. 71.8 %; IISHMS 97.8 vs. 91.4 %, respectively; p < 0.0001).

Younger patients were heavier with age (per year) being inversely correlated with BMI (p < 0.0001) and WC (p < 0.0001). Similar patterns were observed among BMI categories.

The prevalence of prior CVD was similar across all BMI categories (80.1, 80.5, 82.1, 81.9 and 80.8 %, respectively; p = 0.4491) and WC categories for both criteria (ATPIII: 80.9 vs. 82.5 %; IISHMS: 81.1 vs. 82.9 %).

Twelve percent of patients were current smokers, and 46 % were previous smokers. Tables 2 and 3 show that the prevalence of current smokers was higher, and the prevalence of previous smokers was lower in patients with normal BMI and WC when compared with higher BMI and WC categories.

A significant decrease in diabetes duration with increasing degrees of obesity was observed across BMI (p = 0.0001) and WC (p = 0.0044) categories.

Obesity and overweight were more frequent in Caucasian and Black individuals than in Asian or “other” races. Thus, the prevalence of patients with BMI <25 kg/m^2^ among races was highest in Asian people followed by “Other race”, Black and Caucasian people (35.4, 14.0, 6.9 and 5.9 %, respectively; p < 0.0001). Also, the percentage of subjects with healthy WC according to ATPIII and IISHMS was higher in Asian people following the same order as BMI (ATPIII: 54.3, 26.1, 18.0 and 16.6 %; IISHMS: 19.4, 7.2, 6.0 and 4.6 %; p < 0.0001).

Regarding demographic areas, the highest prevalence of overweight and obesity and central adiposity was in the USA, followed by Europe, “Other areas” and Asia (p < 0.0001). Consequently, the prevalence of normal weight in the USA, Europe, “Other areas” and Asia was 4.8, 5.9, 10.6 and 14.0 %, respectively.

Similar patterns of association between obesity (according to BMI and WC categories), age, gender, prior CVD, tobacco use and diabetes duration were observed when different races and ethnicities were analysed separately.

### Cardiometabolic risk factors and medication use

**Fig. 1:**
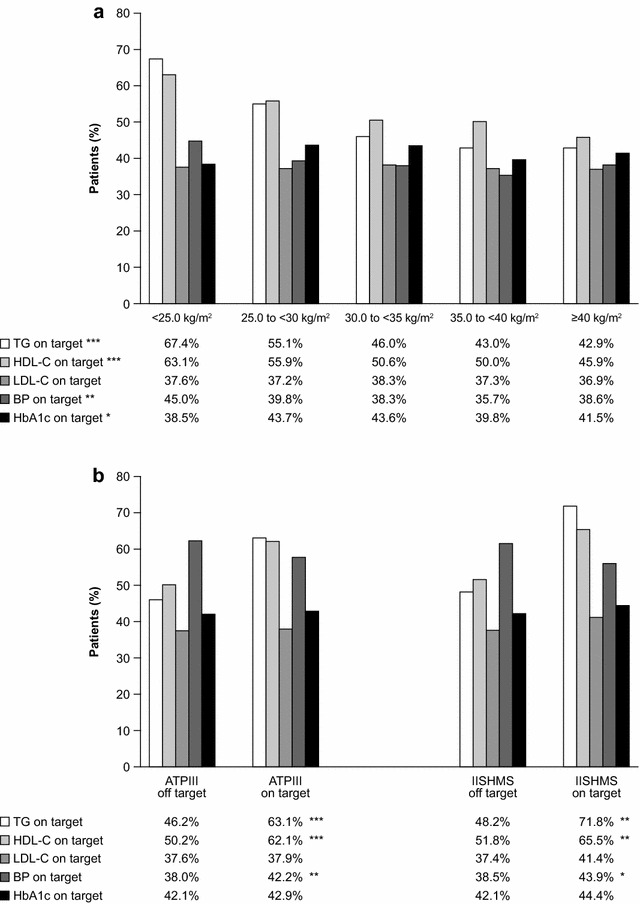
Percentage of patients with cardiometabolic parameters at target among (**a**) BMI and (**b**) WC categories. Statistics. p values of BMI or WC vs. factors were calculated using Chi square test.***p < 0.0001; **p < 0.001; *p < 0.01. ATPIII, adult treatment panel III criteria; BMI, body mass index; BP, blood pressure; HbA1c, glycated haemoglobin; HDL-C, high-density lipoprotein cholesterol; IISHMS, International Joint Interim Statement for the Harmonization of the Metabolic Syndrome criteria; LDL-C, low-density lipoprotein cholesterol; OADs, oral antihyperglycaemic drugs; TG, triglycerides; WC, waist circumference

**Fig. 2:**
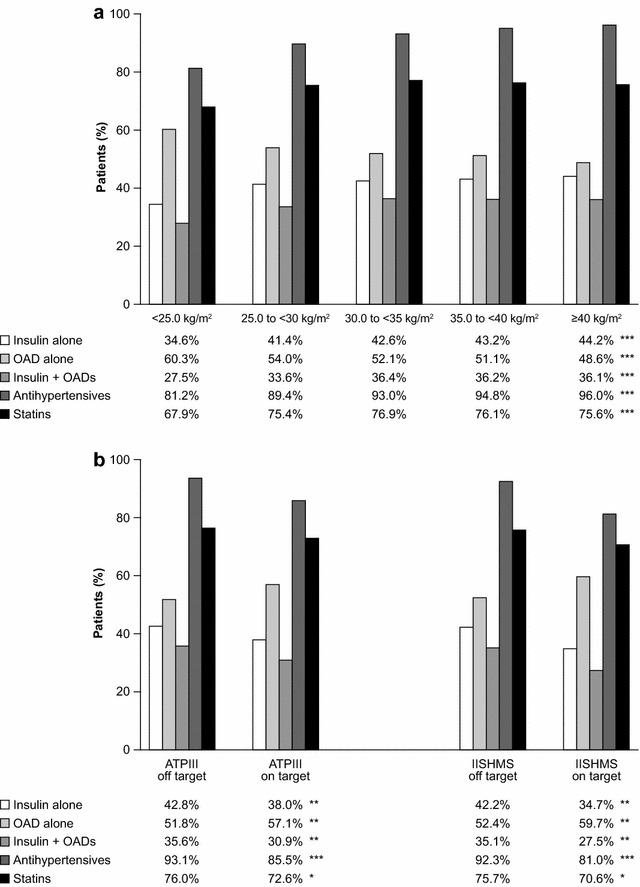
Percentage of patients with relevant medication among (**a**) BMI and (**b**) WC categories. Statistics. p values of BMI or WC vs. factors were calculated using Chi square.***p < 0.0001; **p < 0.001; *p < 0.01. ATPIII, adult treatment panel III criteria; BMI, body mass index; IISHMS, International Joint Interim Statement for the Harmonization of the Metabolic Syndrome criteria; OADs, oral antihyperglycaemic drugs; WC, waist circumference

**Fig. 3:**
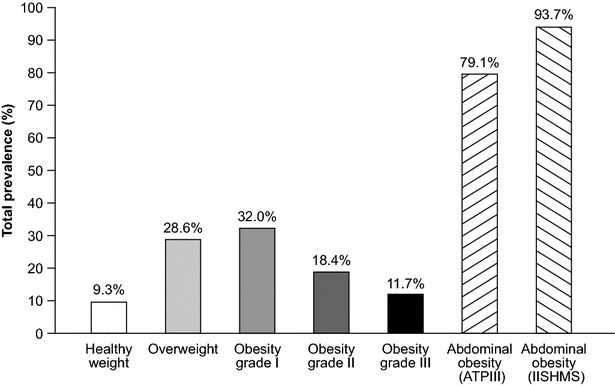
Prevalence of patients who are overweight, obesity grade I, obesity grade II and obesity grade III* and with abdominal obesity. *The sum of the prevalence of patients in BMI obesity grades equal 100 %. ATPIII, adult treatment panel III criteria; BMI, body mass index; IISHMS, International Joint Interim Statement for the Harmonization of the Metabolic Syndrome criteria; WC, waist circumference

Cardiometabolic risk factors and medication use by BMI and WC categories are shown in Tables 2 and 3 and Figs. 1 and 2, respectively. A summary of the prevalence of patients for each BMI and WC class are provided in Fig. 3.

Unadjusted observed values for triglycerides (TG) were positively correlated with BMI (p < 0.0001) and WC (p < 0.0001). HDL-C was negatively correlated with BMI (p < 0.0001) and WC (p < 0.0001). Positive correlations were also found for BP with BMI and WC. No significant correlations were found between HbA1c and BMI or WC.

In the Chi squared analysis (unadjusted), the percentage of patients with target levels of TG, HDL-C and BP dropped significantly with increasing BMI (Fig. 1a). However in the case of HbA1c on target, this trend was reversed; the percentage of patients with HbA1c at target increased significantly with increasing BMI (BMI >25 kg/m^2^) (Fig. 1a). A similar trend was observed for both WC criteria (Fig. 1b).

Obese patients were more likely to be on insulin. The prevalence of patients on insulin was significantly lower in patients with normal weight (Fig. 2a) and normal abdominal waist circumference (Fig. 2b) compared with higher BMI and WC categories. There was a higher percentage of patients on OADs plus insulin at higher BMI (>25 kg/m^2^) and off-target WC categories (Fig. 2).

In the same way, obese patients were more likely to be treated with statins and antihypertensive medication than those of normal weight. The prevalence of patients on these drugs was greater at higher BMI (Fig. 2a) and WC (Fig. 2b) categories. Also, patients with higher BMI (>25 kg/m^2^) were taking a greater number of antihypertensive agents (p < 0.0001). Despite the lipid-modifying therapy and higher numbers of antihypertensive drugs, the lipid profile and BP were worse in obese patients than in those of normal weight. On the contrary, no difference in glycaemic control (HbA1c) was found between BMI and WC categories.

### Multivariable logistic regressions modelling BMI and WC

Multivariable binary logistic regression models using BMI ≥30 kg/m^2^ and off-target WC (using IISHMS and ATPIII) as dependent variables, and relevant demographic, clinical, cardiometabolic and medication parameters as independent variables, were performed in order to elucidate which variables were more related with being obese when having T2DM (Table 4). These analyses are adjusted data, providing the overall relationship of factors with obesity, and therefore supersede the unadjusted data.

**Table 4:** An overview of multivariable logistic regression: baseline characteristics associated with obesity (BMI ≥ 30 kg/m^2^) and increased waist circumference (WC-ATPIII and WC-IISHMS) off target

	BMI ≥30 kg/m^2^			WC-ATPIII off target			WC-IISHMS off target		
	Odds ratio	95 % CI for OR	p value	Odds ratio	(95 % CI for OR)	p value	Odds ratio	(95 % C.I. for OR)	p value
Age (per year)	0.956	(0.949–0.964)	<0.0001	0.998	(0.988–1.007)	0.6461	1.005	(0.990–1.021)	0.5158
Gender									
Male	0.597	(0.525–0.678)	<0.0001	0.136	(0.112–0.164)	<0.0001	0.190	(0.137–0.259)	<0.0001
Female	1.000	(1.000–1.000)	–	1.000	(1.000–1.000)	–	1.000	(1.000–1.000)	–
Smoking status									
Current smoker	0.612	(0.517–0.724)	<0.0001	0.627	(0.516–0.764)	<0.0001	0.604	(0.453–0.813)	0.0007
Never smoked	0.927	(0.821–1.046)	0.2189	0.797	(0.689–0.922)	0.0022	0.911	(0.724–1.148)	0.4295
Previous smoker	1.000	(1.000–1.000)	–	1.000	(1.000–1.000)	–	1.000	(1.000–1.000)	–
Region									
Asia	0.207	(0.137–0.314)	<0.0001	0.361	(0.240–0.543)	<0.0001	0.358	(0.199–0.625)	0.0004
Europe	0.500	(0.430–0.579)	<0.0001	0.685	(0.569–0.823)	0.0001	0.901	(0.659–1.227)	0.5106
Other areas	0.495	(0.426–0.576)	<0.0001	0.646	(0.536–0.777)	<0.0001	0.683	(0.504–0.925)	0.0140
United States	1.000	(1.000–1.000)	–	1.000	(1.000–1.000)	–	1.000	(1.000–1.000)	–
Race									
Asian	0.226	(0.159–0.317)	<0.0001	0.240	(0.168–0.341)	<0.0001	0.473	(0.290–0.803)	0.0039
Black	0.663	(0.545–0.808)	<0.0001	0.628	(0.493–0.804)	0.0002	0.690	(0.474–1.020)	0.0565
Other	0.598	(0.465–0.771)	0.0001	0.699	(0.521–0.944)	0.0180	0.782	(0.482–1.324)	0.3368
Caucasian	1.000	(1.000–1.000)	–	1.000	(1.000–1.000)	–	1.000	(1.000–1.000)	–
Ethnicity									
Hispanic or latino	0.528	(0.446–0.625)	<0.0001	0.545	(0.445–0.668)	<0.0001	1.121	(0.790–1.620)	0.5311
Not hispanic or latino	1.000	(1.000–1.000)	–	1.000	(1.000–1.000)	–	1.000	(1.000–1.000)	–
HbA1c (mmol/mol)	0.999	(0.995–1.002)	0.4850	0.999	(0.995–1.003)	0.7628	0.995	(0.989–1.002)	0.1402
Diabetes duration per year	0.983	(0.977–0.990)	<0.0001	0.981	(0.973–0.989)	<0.0001	0.982	(0.970–0.995)	0.0051
Hypertension (>140/80 mm Hg)									
No	0.779	(0.696–0.871)	<0.0001	0.862	(0.754–0.984)	0.0281	0.862	(0.701–1.062)	0.1623
Yes	1.000	(1.000–1.000)	–	1.000	(1.000–1.000)	–	1.000	(1.000–1.000)	–
Blood lipids									
LDL-C (mmol/L)	0.903	(0.848–0.963)	0.0018	0.914	(0.845–0.988)	0.0234	1.029	(0.907–1.170)	0.6590
HDL-C (mmol/L)	0.620	(0.511–0.752)	<0.0001	0.516	(0.411–0.646)	<0.0001	0.416	(0.303–0.574)	<0.0001
Triglycerides (mmol/L)	1.278	(1.192–1.370)	<0.0001	1.417	(1.298–1.548)	<0.0001	1.680	(1.438–1.974)	<0.0001
eGFR (mL/min/1.73 m^2^)	0.998	(0.996–1.000)	0.0209	1.001	(0.998–1.003)	0.6185	1.001	(0.997–1.005)	0.5649
Albumin-creatinine ratio (per doubling)	0.994	(0.971–1.017)	0.5823	1.016	(0.989–1.044)	0.2459	1.012	(0.970–1.055)	0.5866
Number of antihypertensive drugs									
0	0.306	(0.217–0.428)	<0.0001	0.320	(0.207–0.483)	<0.0001	0.364	(0.175–0.693)	0.0037
1	0.558	(0.411–0.751)	0.0002	0.543	(0.364–0.790)	0.0020	0.757	(0.374–1.390)	0.4013
2	0.599	(0.443–0.801)	0.0007	0.615	(0.415–0.890)	0.0125	0.686	(0.343–1.244)	0.2484
3	0.862	(0.627–1.175)	0.3546	0.860	(0.567–1.277)	0.4659	1.217	(0.580–2.361)	0.5793
4	1.000	(1.000–1.000)	–	1.000	(1.000–1.000)	–	1.000	(1.000–1.000)	–
Pretreatment									
Insulin + OADs	1.298	(1.153–1.463)	<0.0001	1.281	(1.110–1.480)	0.0007	1.525	(1.213–1.926)	0.0003
Insulins use	1.022	(0.826–1.266)	0.8418	1.040	(0.808–1.345)	0.7625	1.196	(0.804–1.830)	0.3931
None/diet	1.297	(1.018–1.660)	0.0372	1.174	(0.881–1.582)	0.2821	1.208	(0.779–1.949)	0.4189
OADs use	1.000	(1.000–1.000)	–	1.000	(1.000–1.000)	–	1.000	(1.000–1.000)	–

The full list of variables used were age, sex, smoking status, region, race, ethnicity, CVD stratum, diabetes duration, HbA1c, hypertension, number of antihypertensive drugs, eGFR, LDL-C, HDL-C, triglycerides, albumin/creatinine ratio, statin use, hyperlipidaemia, aspirin use and number of previous antihypertensive agents. *ATPIII* Adult treatment panel III criteria; *BMI* body mass index; *eGFR* estimated glomerular filtration rate; *HbA1c* glycated haemoglobin; *HDL-C* high-density lipoprotein cholesterol; *IISHMS* International Joint Interim Statement for the Harmonization of the Metabolic Syndrome criteria; *LDL-C* low-density lipoprotein cholesterol; *OADs* oral antihyperglycaemic drugs; *SD* standard deviation; *WC* waist circumference

As shown in Table 4, binary logistic regressions revealed that being obese (BMI ≥30 kg/m^2^) was significantly associated with being younger, female, previous smoker (vs. current smoker), Caucasian (vs. Asian, Black or other races), not Hispanic or Latino (vs. not Hispanic or Latino), from the USA (vs. Asia, Europe, or other areas), shorter diabetes duration, having uncontrolled BP, reduced estimated glomerular filtration rate (eGFR), antihypertensive drug intake (four antihypertensive drugs vs. zero, one or two antihypertensive drugs), insulin plus OAD treatment (vs. OADs only), high levels of TG and lower levels of LDL-C and HDL-C.

Regarding WC, abdominal obesity according to ATPIII was significantly and positively related to the same variables as BMI ≥30 kg/m^2^, with the exception of age, reduced eGFR and no pre-treatment/diet only. In contrast, when the IISHMS were applied, abdominal obesity was mainly related to being female (vs. male), previous smoker (vs. current smokers), Caucasian (vs. Asian), from the USA (vs. Asia or other areas), antihypertensive drug intake (four antihypertensive drugs vs. zero antihypertensive drugs), insulin plus OAD treatment (vs. OADs only), higher levels of TG and lower levels of HDL-C. The associations for hypertension and antihypertensive drug intake were less significant when considering IISHMS compared to ATPIII indicating that, at least for BP, the larger the abdominal circumference, the larger the cardiometabolic impact. There was no statistically significant relationship between HbA1c and the obesity criteria evaluated. Likewise, despite the relationship between eGFR and BMI, no correlation was determined between the albumin–creatinine ratio and BMI or WC criteria.

## Discussion

Our principal finding is that in this very high-risk population of patients with T2DM, the prevalence of overweight (28.6 %) and obesity (61.7 %) is very high. Only 9.1 % of the patients were of normal weight. Likewise, only 20.9 and 6.3 % of patients had a healthy WC according to the ATPIII and IISHMS for metabolic syndrome, respectively. The baseline data from the LEADER trial gave us the opportunity to study the prevalence of overweight and obesity in a high CV risk population with T2DM, and also the association with a set of cardiometabolic risk factors and their treatment intensity.

Worldwide, the proportion of adults with BMI ≥25 kg/m^2^ is estimated to be 36.9 % in men and 38.0 % in women. These figures range from 20.2–22.5 % in South Asia to 70.3 and 60.5 % in high-income North America [17]. In the USA, data from the National Health and Nutrition Examination Survey (NHANES) 2005–2010 indicate a prevalence of patients with T2DM with a BMI ≥25 kg/m^2^ to be 87.1 % (BMI >30 kg/m^2^: 61.2 %) [18]. In high-risk patients with T2DM, data from the Bypass angioplasty revascularization investigation in type 2 diabetes (BARI-2D) trial in patients with T2DM and documented coronary artery disease showed the prevalence of obesity (BMI ≥30 kg/m^2^) to be 56.4 % [19]. Similarly, in the saxagliptin assessment of vascular outcomes recorded in patients with diabetes mellitus (SAVOR)–thrombolysis in myocardial infarction (TIMI) 53 study, the prevalence of obesity (BMI ≥30 kg/m^2^) was 53 % [20].

An unexpected and novel finding was an inverse correlation between diabetes duration and both BMI and WC. Recently, an inverse correlation between BMI strata, diabetes duration and age has also been reported in a pooled analysis of cross-sectional data from Spanish patients with a mean age of 63.2 years [21]. This inverse relationship could suggest less advanced disease and/or the absence of comorbidities in patients with a higher BMI, which could be related to a higher survival probability in overweight patients with T2DM or previous CV disease in prospective studies [22]. The negative association between disease duration and baseline BMI could, therefore, be due to survivor bias: the patients with persistently high BMI would have a greater likelihood of dying before study enrolment than those with lower BMI.

In our study, obesity was more prevalent in younger patients, women, Caucasians, “non-Hispanic or Latinos”, and previous smokers when compared with corresponding groups. These observations seem to be consistent with data from the BARI-2D, a trial that featured a comparable population regarding age and BMI [19].

The higher BMI and lower WC observed in females compared with men are in line with previous findings that men have greater levels of visceral fat compared with women; thus, T2DM and CV disease may develop at a lower BMI level in men than in women [23, 24]. Also, the higher percentage of individuals of normal weight and with a healthy WC among Asian and Hispanic high-risk patients, even according to IISHMS, indicates that these individuals would have developed T2DM and have a higher CV risk at lower BMI and WC. Accordingly, the recent cross-sectional study from the UK Biobank Participants (which included 490,288 subjects) concludes that obesity should be defined by a lower threshold in non-Caucasian than in Caucasian populations [10]. For instance, for T2DM diagnosis, a BMI of 30 kg/m^2^ in Caucasians equates to 22 kg/m^2^ in South Asians, and for respective WCs, 102 cm equates to 79 cm [10].

### Obesity and cardiometabolic risk factors

In common with other studies, we did not find an association between weight and HbA1c level through our logistic analysis [19, 21, 25]. Conversely, we did observe an association between BMI and WC and several cardiometabolic risk factors, despite the more frequent use of statins and antihypertensives in higher BMI and WC categories. Primarily, obesity was associated with higher BP levels, higher levels of TG and low levels of HDL-C. This association was observed both across the trial population and after adjustment for potential confounding factors that are also related to obesity. These results are generally consistent with data from observational studies both in the general population and in high CV risk patients with T2DM [3, 20, 21, 25–28]. Also, a significant association of reduced eGFR with BMI (but not WC) was observed, consistent with kidney dysfunction data from the BARI-2D trial [19].

### Obesity and treatment intensity

When we analysed the influence on lipid levels, the numbers of patients treated with lipid-modifying agents increased with increasing categories of BMI and WC. However, obese patients were less likely to be at the defined lipid targets, especially for HDL-C and TG levels. While the association between statin treatment and BMI is quite consistent in the medical literature [19, 26, 29–31], a greater discrepancy exists around the probability of LDL-C reaching target levels [26, 30].

The percentage of patients not receiving statins at baseline was 24.7 %. Similarly, the baseline prevalence of patients not treated with statins has been reported to be 20 % in the TECOS trial (TECOS; trial evaluating CV outcomes with sitagliptin) [32]. It is also a concern that a high percentage of patients with T2DM with normal adiposity measurements are not treated with statins, despite clinical practice guidelines recommending that high-risk patients with T2DM should be treated with a statin regardless of lipid levels [33, 34]. The latter is based on data showing that statins may have cardio-protective effects extending beyond their cholesterol-lowering properties [35]. Similarly, patients with normal weight were less likely to be treated with antihypertensive agents compared to overweight patients, even though, similar to statins, some antihypertensive agents have shown vascular protective properties [36]. This treatment disequilibrium between lean and obese patients could contribute to the so-called “obesity paradox” [3].

Finally, analyses of other large randomised trials indicate that a significant proportion of patients with T2DM and coronary artery disease fail to achieve pre-specified targets for the major modifiable CV risk factors, suggesting that a combination of barriers may be preventing goals attainment [37]. Our results show that this gap is larger in obese patients with T2DM despite more intensive treatment. It is worth remembering, however, that we present data available at the time after end of the recruitment. The Standard of Care Guidelines for LEADER, developed by the Global Expert Panel, recommended statins for all patients and the following targets: HbA1c <7.0 % (53.0 mmol/mol) (individualised depending on the patient), LDL-C <2.6 mmol/l (<1.8 mmol/l in patients with previous CV disease) and BP 130/80 mmHg [7]. Therefore, a cardiometabolic improvement for all participants regardless of treatment allocation could be observed during the trial.

### Strengths and limitations

Strengths of our study include the large number of patients studied from a multinational and multi-ethnic population, and the context of a clinical trial where a central laboratory was used. The investigators collected data according to a standard protocol. Furthermore, taking into account not only BMI but also WC as a more accurate measure of visceral adiposity is a strength compared with previous studies. Finally, our sample allowed us to study two high-risk groups of patients: a cohort with prior CVD and a high-risk cohort but without prior CVD. Also, the multiracial population potentially makes our data more generalisable.

In interpreting our findings, however, several important limitations need to be considered. The context of a clinical trial may generate a selection bias and diminish the generalisability of the results. Thus, given the known effect of liraglutide to promote weight loss [38], overweight and obesity may be over-represented in LEADER. Also, the observation that only patients >50 years were enrolled in LEADER is important because some studies suggest that the relationship between BMI and CV risk could be modified by age; thus, the findings may not be generalisable to a younger population. In particular, a prospective analysis of individual records of 221,934 people indicated that the increased risk of CVD (myocardial infarction and ischaemic stroke) associated with BMI and WC was 3–4 times higher at the age of 40–59 years than at 70 years [39]. Nevertheless, post hoc analyses of the SCOUT trial that recruited patients ≥55 years confirmed a relationship between weight loss and CV risk factors (such as total cholesterol) and outcomes (such as CV mortality) [40, 41]. Furthermore, although including patients <50 years, studies have shown an association of obesity with suboptimal control of CV risk factors in diabetic patients across the mean age range of 62–65.7 years [2, 19, 21, 28].

Another limitation to these findings is the two distinct cohorts used in this study. Female gender, for example, showed lower prevalence in the patients with prior CVD cohort than in those without prior CVD (33.5 vs. 45.4 %, respectively); there were also large differences in prevalence between patients with prior CVD and those without in patients aged 50–59 years (30.2 vs. 1.5 %, respectively), aged 60–69 years (46.1 vs. 76.5 %, respectively) and those using hypertensive medication (6.8 vs. 15.7 %, respectively).

A further limitation is the cross-sectional nature of the study that precludes determination of causality. Furthermore, although our analyses are adjusted for multiple variables, these might have different effects in longitudinal studies. In addition, the use of single baseline values for blood pressure, cholesterol, and markers of glycaemia has been criticised because of their potential for error in measurement and within-patient fluctuation [42].

Unfortunately, detailed information about the drug classes, doses and treatment adherence were not available at the time this manuscript was developed, and therefore, the authors could not accurately evaluate the association between adiposity and treatment intensity. Finally, we did not have data on risk-taking behaviours such as unhealthy diet, sedentary lifestyle, and alcohol intake, among others, that could confound the relationship between adiposity and attainment of treatment target.

When entering the treatment phase, an important aspect is the demonstrated beneficial effects of GLP-1RA on weight. Exenatide once weekly has been, at least partly, associated with weight loss and reduced macrovascular risk in a retrospective study and a pooled analyses of eight studies [43, 44]. A retrospective longitudinal pharmaco-epidemiological study established that the risk for composite of myocardial infarction or stroke in overweight and obese patients were not significantly higher compared to those in the normal weight group after adjusting for other factors [43]. Also, the rates/1000 person years of individual CV events in the same study were not significantly different by BMI categories (normal weight, overweight and obese) regardless of treatment groups in the study cohort [43]. Moreover, a pooled analysis from eight studies of exenatide once weekly showed that the greatest trend of improvement in CV risk factors was observed in patient quartiles with the greatest reductions in body weight [44]. Following the same relationship as exenatide, liraglutide has demonstrated beneficial changes in weight and body composition; indeed, 12 and 56 week trials specifically designed to study the efficacy and safety of liraglutide for weight management have shown reductions in body weight and enhanced metabolic parameters (such as B-type ventricular natriuretic peptide, fasting lipids and urinary albumin-to-creatinine ratio) [45–47].

In summary, overweight and obesity are extremely prevalent in high-risk patients with T2DM. Furthermore, BMI and WC are related to major cardiometabolic risk factors, particularly BP, TG, and HDL-C. In addition, treatment intensity is higher in overweight and obese patients, compared with others; however, the rates of treatment and control of lipids and BP are remarkably suboptimal in overweight and obese individuals. As secondary data, LEADER will explore the longitudinal effects of liraglutide or placebo, when added to standard care, on CV risk factors and weight for up to 5 years of treatment.

## Abbreviations

ATPIII: adult treatment panel III criteria
BARI-2D: bypass angioplasty revascularization investigation in type 2 diabetes
BMI: body mass index
BP: blood pressure
CV: cardiovascular
CVD: cardiovascular disease
eGFR: estimated glomerular filtration rate
GLP-1RA: glucagon-like peptide-1 receptor agonist
HbA1c: glycated haemoglobin
HDL-C: high-density lipoprotein cholesterol
LDL-C: low-density lipoprotein cholesterol
IISHMS: International Joint Interim Statement for the Harmonization of the Metabolic Syndrome criteria
NHANES: national health and nutrition examination survey
OAD: oral antihyperglycaemic drug
T2DM: type 2 diabetes mellitus
TECOS: trial evaluating cardiovascular outcomes with sitagliptin
TG: triglycerides
WC: waist circumference
